# Some of the Causes and Effects of Mouth Breathing1Read at the thirty-second annual meeting of the Medical Association of Central New York, at Syracuse, October 17, 1899.

**Published:** 1900-01

**Authors:** J. M. Ingersoll

**Affiliations:** Rochester, N. Y.


### SOME OF THE CAUSES AND EFFECTS OF MOUTH BREATHING.[1]

BY J. M. INGERSOLL, M. D., Rochester, N. Y.

IT IS not my intention to try to enter into an exhaustive and technical discussion of this subject, both from lack of time and a feeling that such a presentation would be presumptive and out of place during such a busy session as this one will be. I shall try to

1. Read at the thirty-second annual meeting of the Medical Association of Central New York, at Syracuse, October 17, 1899.

present to you some of these forms of obstruction to nasal breathing that are most commonly met, and which the general practitioner is called upon to diagnosticate.

This subject should attract the attention of every practitioner of medicine, because through the family physician many persons may be saved the suffering and deformity due to causes easily removed. The general practitioner owes it to the families he attends, to avert the pernicious effects of obstruction to free nasal respiration in the children growing up under his professional care. Too often is the condition allowed to persist until an evil is created that can never be completely remedied; and I believe that a physician who allows such a condition of things to continue, is derelict in his duty to his patient.

This is especially true of the first cause of mouth-breathing which I shall consider—adenoid growths or vegetations in the nasopharynx. I have known cases of this disease to be neglected because the family physician has said, when consulted about the condition of the child, "Oh, let them alone, the child will outgrow them, and they will disappear." There is just enough truth in this assertion to make it especially dangerous. The child *will* often outgrow them, and they *will* disappear, but, by the time this disappearance has taken place, the child is marked for life.

In children from infancy to the age of seventeen these growths are probably the commonest cause of mouth-breathing, and perhaps the most important of all, because they do occur so frequently in young children. They are, I believe, far commoner than most of us are apt to suspect. They are found in very young, as well as in patients of mature age. Bosworth gives two tables containing analyses of the ages of patients in which these growths were found.

The first is by Meyer, who first called the attention of the profession to these as distinctive growths, showing that the cases ranged in age from under 5 years to 45, and that out of 102 cases, 34 were in patients between 5 and 10 years of age; 25 between 10 and 15 years; 21 between15 and 20 years, and 11 between 20 and 25 years; only three cases occurring in children under 5 years of age. Bosworth's own tabulated cases were 75 in number, of which only 5 were under 10 years, 16 between 10 and 15 years, 27 between 15 and 20 years, and 23 between 20 and 30 years. The others were in patients up to 50 years of age.

In explanation of the difference in the average ages of his and Meyer's patients, Bosworth says that he includes in his tables the cases of broad flat growths occurring in the pharyngeal vaults of adult patients, as he considers them essentially the same as the growths in children. In this I agree with him.

In my own experience I have found a larger proportion of cases in children under 5 years than either of these tables show, and I believe the reason is that the general profession is more observant of these cases than in the past, and has patients treated much earlier in life. The cause of adenoids is still undetermined, but it is observed that they are likely to occur, and are more largely developed in children of "scrofulous diathesis," but all the late writers seem to think that scrofula, *per se*, is not the direct cause. That there is hereditary tendency would seem to be indicated by the frequency of several cases occurring in the same family, with at least one of the parents bearing signs of a similar trouble in former years. The so-called lymphatic tendency is observed in a great number of cases, the faucial tonsils and cervical glands being involved in the hypertrophic condition.

The diagnosis of this condition is easy, especially in persons past the period of inarticulation. The voice alone should lead one to suspect the presence of these growths. There is a "dead" quality to the tones; a lack of resonance, which can be due only to some form of nasal obstruction. The symptom, however, which never should fail to call attention to these parts is mouth-breathing. When you observe this symptom in any of your young patients examine for a nasopharyngeal space filled with adenoids, as this condition is most probably present. The nose is primarily an organ of respiration, and a child will always use it as such, unless there is some mechanical obstruction present, when, of course, mouth-breathing must be resorted to, in which case an examination of the nose and pharynx is imperatively demanded. If the patient is old enough a rhinoscopic examination will reveal the condition, and frequently an examination through the nose will establish the diagnosis. The finger in the nasopharynx is an unfailing index, and should always be used in case of doubt.

Nasal stenosis and consequent mouth-breathing may be caused by the presence of hypertrophied lingual tonsils. These tonsils are usually concurrent with adenoid vegetations, but are frequently present without adenoids. A visible tonsil is always an evidence of diseased action, and when large enough to cause trouble should be attended to. I have entirely relieved several cases of mouth-breathing by the removal of enlarged tonsils. Prominent among cases of obstruction to the nasal passages is that form of growth called polypus. It is a common cause of stenosis, discomfort and pain. The diagnosis is easy as these growths are usually in a position to be plainly seen, having their origin, as a rule, on the second turbinate bone. In any adult, having a characteristic dead, flat voice, "talking through his nose" (which he is not) always suspect the presence of nasal polypi. They are usually bilateral, but occasionally occur in one side only. Other causes of mouth-breathing are spurs of the nasal septum, deviations of the nasal septum, hypertrophies of the turbinated bodies, cysts of the turbinated bodies, especially the second turbinate, rhinoliths, papilloma, and other rarer forms of deformity and disease.

The evil effects of mouth-breathing are many and wide-spread. First, as to its bearing on anatomical development. A person who has been an habitual mouth-breather from childhood presents a very characteristic appearance, due to the interference with development of the facial bones, from non use of the nose in respiration. The face is long, narrow and flat, the nostrils usually mere slits, lower jaw protruding and drooping, giving a stupid expression to the face. On inspection of the mouth, the arch of the hard palate is seen to be very high and narrow, and the teeth in the upper jaw are very irregular, so much so that many adults have had numbers of teeth drawn to "make room," and not a few have been obliged to have all the teeth drawn, and wear a plate, so uncomfortable and unsightly were their own teeth. This comes from the nondevelopment of the superior maxillary, producing what is often called "dish face."

Aside from anatomical defects, the physiological effects are decidedly injurious. The tongue, cheeks, pharynx and larynx are injuriously affected. During respiration the inspired air should be warmed and moistened before reaching the pharynx, larynx and lungs. In normal respiration this object is attained by the passage of the air through the nose, where it is properly prepared for the further use in the lungs. When, however, nasal respiration is impossible, moisture is snatched from the tongue, cheeks, pharynx and larynx, which are not supplied with the proper apparatus to furnish it. As a consequence these structures are kept in a state of chronic inflammation, which aside from any remote injurious effects, causes great discomfort, and often pain to the unfortunate mouth-breather. The mental processes of mouth-breathers are slow. Especially is this noticed in the morning after the unfortunate has spent a night of heavy, but unrefreshing sleep. He awakens, or is awakened, with a decided feeling of drowsiness, and has a well marked case of "that tired feeling," which, however, Hoods' sarsaparilla would never cure. His tongue is parched, cheeks dry, pharynx coated with a thick, sticky mucus, which has given up all the moisture that can be extracted from it. His eyes seem glued together, "just one more nap" seems most desirable, and when compelled to get up, he feels as if his sleep had not been refreshing. In fact, he does not begin to feel "like himself" until he has been up for some time, and gotten his blood into active circulation.

No mouth-breather is healthy. There is no one disease which can be diagnosticated, but there is a feeling of languor, a lack of endurance, often a functional heart trouble, and a general lack of ambition. Vague symptoms are noticed, and are referred to many causes. Backache, the effective coadjutor of quack kidney remedies, is a common sequence of mouth-breathing, due to the lack of rest during sleep. These symptoms usually disappear or grow less marked as the day progresses, and the patient may feel quite well during the afternoon or evening. These bad symptoms are invariably relieved by the removal of the obstruction to nasal breathing and the resumption of the physiological function of the nose.

In addition to the more direct results due to the mouth-breathing of children, in whom adenoids are present, deafness, more or less marked, is commonly noticed. This affliction causes them to become inattentive, and unjustly stamps them with stupidity, when proper attention to their condition, with appropriate treatment, would save them much humiliation. Mention here of the treatment of the various conditions described is hardly necessary. The indication is for the removal of the obstruction, of whatever nature, but a description of the technique would make this paper too long, and take up your time unnecessarily.

